# National Antibiotic Consumption for Human Use in Sierra Leone (2017–2019): A Cross-Sectional Study

**DOI:** 10.3390/tropicalmed6020077

**Published:** 2021-05-13

**Authors:** Joseph Sam Kanu, Mohammed Khogali, Katrina Hann, Wenjing Tao, Shuwary Barlatt, James Komeh, Joy Johnson, Mohamed Sesay, Mohamed Alex Vandi, Hannock Tweya, Collins Timire, Onome Thomas Abiri, Fawzi Thomas, Ahmed Sankoh-Hughes, Bailah Molleh, Anna Maruta, Anthony D. Harries

**Affiliations:** 1 National Disease Surveillance Programme, Sierra Leone National Public Health Emergency Operations Centre, Ministry of Health and Sanitation, Cockerill, Wilkinson Road, Freetown, Sierra Leone; 2 Department of Community Health, Faculty of Clinical Sciences, College of Medicine and Allied Health Sciences, University of Sierra Leone, Freetown, Sierra Leone; 3 Special Programme for Research and Training in Tropical Diseases (TDR), World Health Organization, 1211 Geneva, Switzerland; khogalim@who.int; 4 Sustainable Health Systems, Freetown, Sierra Leone; hann.katrina@gmail.com (K.H.); bmollehshs@gmail.com (B.M.); 5 Unit for Antibiotics and Infection Control, Public Health Agency of Sweden, Folkhalsomyndigheten, SE-171 82 Stockholm, Sweden; wenjing.tao@fohm.se; 6 Pharmacy Board of Sierra Leone, Central Medical Stores, New England Ville, Freetown, Sierra Leone; sbarlatt2000@yahoo.com (S.B.); jpkomeh@pharmacyboard.gov.sl (J.K.); jbjohnson@pharmacyboard.gov.sl (J.J.); memosesay@yahoo.com (M.S.); otabiri@pharmacyboard.gov.sl (O.T.A.); thomasfawzi5@gmail.com (F.T.); 7 Department of Pharmaceutics and Clinical Pharmacy & Therapeutics, Faculty of Pharmaceutical Sciences, College of Medicine and Allied Health Sciences, University of Sierra Leone, Freetown, Sierra Leone; 8 Directorate of Health Security & Emergencies, Ministry of Health and Sanitation, Sierra Leone National Public Health Emergency Operations Centre, Freetown, Sierra Leone; mohamedavandi69@yahoo.com; 9 The Lighthouse Trust, Kamuzu Central Hospital, Lilongwe P.O. Box 149, Malawi; hannock.tweya@gmail.com; 10 International Union Against Tuberculosis and Lung Disease, 75006 Paris, France; collins.timire@theunion.org (C.T.); adharries@theunion.org (A.D.H.); 11 Department of Pharmacology and Therapeutics, Faculty of Basic Medical Sciences, College of Medicine and Allied Health Sciences, University of Sierra Leone, Freetown, Sierra Leone; 12 Department of Medical Microbiology, Faculty of Basic Medical Sciences, Faculty of Pharmaceutical Sciences and Faculty of Nursing, College of Medicine and Allied Health Sciences, University of Sierra Leone, Freetown, Sierra Leone; asankohhughes@yahoo.co.uk; 13 WHO Country Office, Freetown, Sierra Leone; marutaa@who.int; 14 London School of Hygiene and Tropical Medicine, London WC1E 7HT, UK

**Keywords:** ATC classification, AWaRe, antibiotic consumption, DDD per 1000 inhabitants per day, AMR surveillance, operational research, SORT-IT, Pharmacy Board of Sierra Leone (PBSL), Essential Medicines List, National Standard Treatment Guidelines

## Abstract

Monitoring antibiotic consumption is crucial to tackling antimicrobial resistance. However, currently there is no system in Sierra Leone for recording and reporting on antibiotic consumption. We therefore conducted a cross-sectional study to assess national antibiotic consumption expressed as defined daily dose (DDD) per 1000 inhabitants per day using all registered and imported antibiotics (categorized under the subgroup J01 under the anatomical and therapeutic classification (ATC) system) as a proxy. Between 2017–2019, total cumulative consumption of antibiotics was 19 DDD per 1000 inhabitants per day. The vast majority consisted of oral antibiotics (98.4%), while parenteral antibiotics made up 1.6%. According to therapeutic/pharmacological subgroups (ATC level 3), beta-lactam/penicillins, quinolones, and other antibacterials (mainly oral metronidazole) comprised 65% of total consumption. According to WHO Access, Watch, and Reserve (AWaRe), 65% of antibiotics consumed were Access, 31% were Watch, and no Reserve antibiotics were reported. The top ten oral antibiotics represented 97% of total oral antibiotics consumed, with metronidazole (35%) and ciprofloxacin (15%) together constituting half of the total. Of parenteral antibiotics consumed, procaine penicillin (32%) and ceftriaxone (19%) together comprised half of the total. Policy recommendations at global and national levels have been made to improve monitoring of antibiotic consumption and antibiotic stewardship.

## 1. Introduction

Antimicrobial resistance (AMR)—the ability of microbes to evolve and withstand the effects of antimicrobials—is a significant cause of morbidity and mortality globally [1,2,3]. So far, AMR has been the reason for 700,000 deaths per year worldwide and the figure is expected to reach 10 million per year by 2050 [4]. In addition to the health implications, the cost of health care for patients with resistant bacteria is higher than the cost of health care for non-resistant infections because of the longer duration of illness, the need for additional tests and more expensive alternative medicines [5]. Thus, AMR does not only hinder our ability to treat infections, but has a substantial economic and societal impact that rolls back the health gains obtained from antimicrobials during the last eight decades and jeopardizes the gains towards achieving the sustainable development goals (SDGs) [6,7]. The impact of AMR is disproportionately higher in low-and middle-income countries (LMICs) which have a higher burden of infectious diseases and often lack the infrastructure, human and financial resources to adequately tackle drug-resistant epidemics [6].

The main driver of the insurgence and spread of AMR is the inappropriate use and consumption of antibiotics. This includes irrational prescribing (for example, inappropriate type, dosage, route of administration, or duration of antibiotic treatment) and over-the-counter sales of antibiotics [8,9]. The association between antibiotic-resistant bacteria and level of antibiotic use is well established, meaning that a reduction in unnecessary consumption would influence and potentially reduce resistance [10,11,12]. Global consumption of antibiotics has increased by 67% between 2000 and 2015 [13], and it is estimated that almost half of all antibiotics used in human medical care are considered inappropriate [9]. Despite the increase in antibiotic consumption worldwide, access to antibiotics is also a continuing challenge. Each year, more than a million children with untreated infections, such as pneumonia and sepsis, die because of lack of or delayed access to antibiotics. In fact, limited or delayed access to antibiotics kill more people than the impacts of antibiotic resistance [2]. In addition, poor access to antibiotics can result in drug resistance, especially in LMICs or their sub-regions [14]. In such settings, patients may not be able to afford a complete course of antibiotics or may only be able to obtain drugs that are suboptimal or falsified, or ones to which the organism is already resistant [2,15].

To preserve the effectiveness of antibiotics and to continue to reap their benefits for health, achieving a balance between prudent use and improved access is key. To this end, the World Health Organization (WHO) has launched a global action plan (GAP) on AMR. One of the main objectives of the GAP is to optimize the use of antimicrobials, including antibiotics [15]. To guide the optimization process, WHO has categorized antibiotics into three categories as AWaRe: Access, Watch and Reserve. The Access category includes first- and second-line antibiotics for empirical treatment needed for common infections, and these should be available in all health care settings. WHO specifies a country-level target of at least 60% of antibiotic consumption being from medicines in the Access category. The Watch category includes antibiotics with a higher potential to develop resistance, and their use as first and second choice treatment should be limited. The Reserve category includes “last resort” antibiotics whose use should be reserved for special settings and multidrug-resistant bacterial infections where alternative treatments have failed [16].

WHO has urged all member states to adapt the GAP to national contexts, and establish a stewardship program that monitors and promotes optimization of antibiotic use [15]. In response to this, Sierra Leone has developed the National Strategic Plan for Combatting AMR in Sierra Leone which aims to monitor antimicrobial consumption at national and local levels and promote optimized use [17]. However, currently there is no system in Sierra Leone for recording and reporting on consumption of antibiotics. Ideally, assessing levels of antibiotic use should rely on data collected at the patient level. However, such data is not available in many LMICs. Such information is necessary to establish a baseline for the strategic plan and serve as a yardstick for monitoring antibiotic consumption over time. Moreover, information on the rates of antibiotics consumed according to the AWaRe categories can inform target-setting and identify inequalities in access to antibiotics.

In 2016, WHO recommended a methodology for antimicrobial consumption surveillance using a standardized population-based metric of defined daily dose (DDD) per 1000 inhabitants [18]. The methodology also includes the assessment of consumption based on aggregated data sources, such as import and sales data, as a proxy for antimicrobial use at the patient level [18].

We therefore carried out this study to assess national antibiotic consumption in Sierra Leone between 2017–2019. Specific objectives of the study were, for the period of 2017–2019, to report on, per route of administration, (i) consumption of antibiotics in defined daily dose (DDD) standardized by population size (DDD per 1000 inhabitants per day) alone and stratified by pharmacological subgroup and AWaRe categorization; and (ii) the top antibiotics consumed at the substance level.

## 2. Materials and Methods

### 2.1. Study Design

This was cross-sectional analysis of routinely collected data on antibiotics imported for human consumption and registered by the Pharmacy Board of Sierra Leone (PBSL).

### 2.2. Setting

#### 2.2.1. General Setting

Sierra Leone is a country on the southwest coast of West Africa bordered by Liberia to the southeast and Guinea to the northeast. The country has a tropical climate, with a diverse environment ranging from savanna to rainforests. Sierra Leone occupies an area of about 71,740 square kilometers with a total population size of 7,075,641 [19]. Administratively, the country is divided into five regions, which are further subdivided into sixteen districts.

The country frequently suffers from disease outbreaks such as Lassa fever, measles, malaria, cholera, yellow fever, meningitis and, more recently, the largest outbreak of Ebola virus disease in 2014–2016 [20].

#### 2.2.2. Specific Setting

##### Health System in Sierra Leone

Sierra Leone has a three-tier health care system of primary, secondary, and tertiary health care. The overall responsibility of managing and organizing health care services is under the Ministry of Health and Sanitation (MoHS). Sierra Leone is divided into 16 health districts that correspond to the administrative districts. Each health district is supported by a health management team which is responsible for planning, organizing and monitoring health provision, training personnel, working with communities and supplying equipment and drugs [21]. Each district supports a district hospital and, on average, 50 peripheral health units. There are four regional referral hospitals and a national referral hospital in the capital provides tertiary care [21]. Health care services are largely provided by the public sector, with some services provided by the private sector, non-governmental organizations and faith-based organizations.

Except for the Free Health Care Initiative for children under five years, pregnant and lactating mothers, drugs are mainly acquired through out-of-pocket purchase. The public sector has a weak cost recovery of drug sales; patients are required to purchase from private pharmacies if prescribed drugs are out-of-stock or not included in the list of drugs procured by the hospital. There is a National Essential Medicines List and Standard Treatment Guidelines for some infectious diseases, but strict adherence to these guidelines is still a big challenge [22]. Microbiology laboratory capacity to guide antibiotic prescribing is limited to tertiary and regional hospitals.

##### Drug Procurement

For the public sector, drugs are procured through contracted suppliers and distributed by the Directorate of Pharmaceutical Services through the Central Medical Stores. Drugs are distributed to government health facilities by both the “pull” and “push” systems (central level down to district and health facilities). Frequent drug stock-outs are common in the public sector [23], meaning that many patients have to buy medicines from outside pharmacies. In the private sector, drugs are purchased from licensed pharmaceutical outlets, which in turn buy drugs from licensed pharmaceutical importers. As there are no drug manufacturing companies in Sierra Leone, all drugs used in the country are imported.

##### Pharmacy Board of Sierra Leone (PBSL) and Drug Regulations

The PBSL, which is directly under the MoHS, was established by The Pharmacy and Drugs Act, 2001 [24] and regulates all pharmaceuticals in the country. PBSL has nine technical departments: Drug Evaluation & Registration; National Pharmaceutical Quality Control Laboratory; Policy Standards & Practice; Complementary & Alternative Medicines; Factory Inspectorate & Import Control; Enforcement & Narcotic Control; Pharmacovigilance & Clinical Trials; Distribution Chain Inspection; and Quality Assurance departments. According to the Pharmacy and Drugs Act, all drugs imported into Sierra Leone have to be registered by licensed importers, and importation is made only after obtaining Import Permits and Clearance Permits [24]. Drug donations also require Import and Clearance Permits. Quality control testing of all pharmaceuticals is mandatory for all drugs registered and imported into the country. However, as Sierra Leone shares porous borders with Guinea and Liberia, illegal imports and exports of medicines are sometimes possible. The Factory Inspectorate and Import Control Department of the PBSL documents all drugs imported into Sierra Leone through the legal channels.

### 2.3. Study Population and Periods

The study population included all registered and imported antibiotics categorized under the subgroup J01 (antibacterials for systemic use) of the Anatomical Therapeutic Chemical (ATC) Classification System, including parenteral and oral formulations (see Section 2.6.1. for explanation) [25]. Nitroimidazole derivatives for protozoal diseases, containing oral metronidazole and tinidazole, were also included as per the WHO methodology for surveillance of antimicrobial consumption and reported under the J01X subgroup, where the parenteral formulations of these substances are classified. Antibiotics used for local therapy (e.g., topical creams, eye/ear drops) are not included in the WHO surveillance methodology and were thus excluded [26].

The study was conducted between October 2019 and March 2021, and included antibiotic data from January 2017 to December 2019.

### 2.4. Data Variables and Sources of Data

Data variables collected included the following: population by year, name of substance, strength of the active ingredient(s), route of administration, and quantity imported. Specific year of import was not available in the electronic database, and consumption for the entire study period was therefore aggregated.

The data sources were the 2015 Sierra Leone Housing Census Population Projections [27]; PBSL Drug Clearance and Importation Permits Database; and the PBSL Drug Registration Records.

### 2.5. Data Collection and Validation

The Drug Clearance and Importation Permits Database is a Microsoft Access database maintained by the Factory Inspectorate & Import Control Department of the PBSL. Data from the Drug Clearance and Importation Permits Database were exported into Microsoft Excel. The route of administration variable was sourced by cross-referencing batch ID from the paper-based Drug Registration Record, also located at the Factory Inspectorate & Import Control Department of the PBSL. Data collection was done by two research assistants. Ten percent (10%) of the records entered were validated by the principal investigator as a quality assurance process.

All import data collected were transferred by two data clerks into a formatted Microsoft excel template with embedded macros to calculate consumption provided by the WHO. The Principal Investigator reviewed all data in the WHO Excel template for quality control. Review of the data resulted in removal of three import values as they were deemed to have data entry errors in the database. These included two entries of imports for ciprofloxacin and one entry for import of metronidazole. There were some assumptions in interpreting the size, quantities, and strengths of some of the products in the database. These included: bottles of suspension, syrup or solutions were assumed to be 100 milliliters in size; and quantities for ‘strips’ of oral tablets were assumed to reflect quantities of tablets.

### 2.6. Data Analysis

#### 2.6.1. Anatomical Therapeutic Chemical (ATC) Classification System

Substances were categorized based on the ATC classification system [28], version 2019. The ATC system classifies drugs (including antibiotics) according to their anatomical, therapeutic/pharmacological and chemical properties. ATC includes levels of classification at the therapeutic/pharmacological subgroup (level 3, ATC3) and the chemical substance (level 5, ATC5).

#### 2.6.2. Defined Daily Dose (DDD)

Consumption was estimated according to the WHO methodology for surveillance of antimicrobial consumption [18], which utilizes defined daily dose (DDD), the assumed average maintenance dose per day of an antimicrobial substance used for its main indication in adults. DDD per product and time period were calculated using the WHO Excel template.

#### 2.6.3. Defined Daily Dose (DDD) per 1000 Inhabitants per Day

To adjust for population size, antibiotic consumption was presented as number of DDDs per 1000 inhabitants per day [26]. This metric can be roughly interpreted as the number of individuals per 1000 inhabitants on antibiotic treatment per day. We calculated DDDs per 1000 inhabitants per day using the average projected populations of Sierra Leone for the study period (2017–2019) [29].

#### 2.6.4. Access, Watch, and Reserve (AWaRe) Categories

Access, Watch, and Reserve (AWaRe) categories based on the WHO Model List of Essential Medicines were assigned to each antibiotic substance (ATC5) using the latest version [30]. Antibiotics substances that are not included in the WHO Model List of Essential Medicines have not yet been categorized, and are reported as “Other” [16]. 

## 3. Results

### 3.1. Consumption of Antiobiotics (DDD per 1000 Inhabitants per Day) Along with Their Subgroup, Route of Administration and AWaRe Categorisation

Total consumption of antibiotics for systemic use for the period 2017–2019 was 19 DDDs per 1000 inhabitants per day. The vast majority consisted of oral antibiotics (98.4%, 18 DDDs per 1000 inhabitants per day), while parenteral antibiotics made up only 1.6% of all antibiotics consumed (0.3 DDDs per 1000 inhabitants per day). Table 1 presents consumption by therapeutic/pharmacological subgroup (ATC level 3). Of the consumed oral antibiotics, the highest DDDs per 1000 inhabitants were found among other antibacterials, consisting mainly of oral metronidazole; beta-lactam/penicillins; and quinolones. Almost half of the consumed parenteral antibiotics consisted of beta-lactam/penicillins, followed by other beta-lactam antibacterials and other antibacterials.

Table 2 shows antibiotic consumption by WHO AWaRe category by route of administration. During 2017–2019, 65% of all oral antibiotic consumption consisted of Access antibiotics and 31% of Watch antibiotics. The proportion of Access antibiotics was even higher for parenteral antibiotics. No Reserve antibiotics were reported according to the import records. 

### 3.2. Most Commonly Consumed Antibiotic Substances by Route of Administration by Chemical Substance Group

Table 3 presents the ten most commonly consumed oral antibiotics in Sierra Leone. The top ten antibiotics represented 97% of all the antibiotics consumed in the country. Metronidazole (35%) and ciprofloxacin (15%) represented approximately half of all oral antibiotic consumption for the study period.

Table 4 presents the parenteral antibiotics consumed in the country. Procaine benzylpenicillin (32%) and ceftriaxone (19%) constituted over half of all parenteral antibiotic consumption for the study period (51%). 

## 4. Discussion

This is the first study to report on antibiotic consumption in Sierra Leone at the national level. The findings of this study are important as they serve as a baseline for future monitoring of antibiotic consumption in the country.

Consumption over a period of three years (2017–2019) was 19 DDD per 1000 inhabitants per day. The most frequently consumed antibiotics according to therapeutic/pharmacological subgroup were beta-lactam/penicillins, quinolones, and other antibiotics, consisting mainly of oral metronidazole. Antibiotics were consumed orally and parenterally at different rates, with parenteral antibiotic consumption representing less than 2% of total DDD per 1000 inhabitants per day. According to the WHO AWaRe categories, the highest consumption was found among Access antibiotics, followed by Watch antibiotics, with no consumption at all of Reserve antibiotics. According to chemical substance group, metronidazole and ciprofloxacin were the most frequently consumed oral antibiotics, while procaine benzylpenicillin and ceftriaxone were the most commonly used parenteral antibiotics.

The total antibiotic consumption observed for Sierra Leone was slightly higher than, but comparable to, the median antibiotic consumption across 76 countries in 2015: 17.2 DDD per 1000 inhabitants per day [31]. Of note, however, is the fact that sub-Saharan African countries were poorly represented in this study [31]. In 16 Eastern European and Central Asian countries, the DDD per 1000 inhabitants per day in 2015 ranged from 8.0–41.5 with a median of 19.0, similar to what was found in Sierra Leone [32]. The findings in Sierra Leone, however, showed a much lower DDD per 1000 inhabitants per day compared with Tanzania (27.3) in 2016. However, Sierra Leone consumption was higher than the reported DDD per 1000 inhabitants in 2015 for Burkina Faso (13.8), Côte d’Ivoire (10.7), and Burundi (4.5) [16]. However, the results from Burundi were based on public sector data only, which may explain the difference with our findings.

In terms of frequently consumed antibiotics per therapeutic/pharmacological subgroup, our findings were similar to those found in Tanzania. In 2015, quinolones comprised the same proportion (14%) of total consumption by ATC3 in Tanzania [16]. Similarly, in a study based on data on antibiotic imports, J01C Beta-lactam antibacterials/penicillins were also one of the three most common antibiotics consumed in Tanzania between 2017 and 2019, which also included doxycycline, and sulfamethoxazole and trimethoprim [33]. A study in Eastern Europe and Central Asia found beta-lactam/penicillins (ATC group J01C) as the most commonly used antibiotics across most countries, and there was also a high consumption of quinolones, similar to this study [32]. The high consumption of quinolones may likely be due to the high burden of clinically diagnosed typhoid fever and sexually transmitted diseases [34]. The high consumption of metronidazole in country is likely to be due to its common use in treating pelvic and gastrointestinal infections as well as septic wounds. Quinolones and metronidazole are accessible and cheap antibiotics, and are effective against a broad range of conditions. They constituted half of all oral antibiotics used in Sierra Leone in our study; it will, therefore, be important to monitor resistance to these antibiotics regularly to guide individual and empirical treatment.

We found that oral antibiotic consumption was 60 times higher than parenteral consumption. The higher proportion of oral antibiotics consumed is comparable to findings from Tanzania, Burkina Faso, and Côte d’Ivoire in 2015 were oral antibiotics represented between 96–98% of consumption of total antibiotics [16]. This is also similar to what was found in Vietnam, where 92% of antibiotic DDD per 1000 inhabitants per day was by oral administration [35]. In settings such as Sierra Leone, oral antibiotics are more available and more easily administered, particularly in the outpatient and community settings. Parenteral administration requires health facility access, and, particularly, inpatient care.

In Sierra Leone, Access antibiotics accounted for 65% of total consumption, comparable to 61% in the 76-country analysis [12], but less than the 90% found in Tanzania [33]. This finding is encouraging, as Sierra Leone is in line with the WHO’s country-level target of at least 60% or more of total antibiotic consumption consisting of Access antibiotics [16].

We found no consumption in the Reserve category of antibiotics in Sierra Leone. This is not an unexpected finding. According to surveillance data from 2015, none of the four sub-Saharan African countries reported any consumption of Reserve antibiotics [16]. While it is recommended that Reserve antibiotics be restricted to last -resort use, these antibiotics should be available in countries and the zero consumption may highlight an access issue. Possible reasons for this may include: (i) Reserve antibiotics are expensive for both importers and patients in the country; (ii) the drugs are not on Sierra Leone’s Essential Medicine List [22]; (iii) import records do not capture the informal market and may miss Reserve antibiotics that enter the country by other means; and (iv) limited laboratory capacity for antibiotic sensitivity testing, which may force clinicians to avoid prescribing second- and third-line antibiotics [36]. As the balance between rational use and access to antibiotics, including the Reserve category, is key to preventing AMR, further research is needed to understand the reasons for zero consumption of this category in Sierra Leone.

This study has several strengths. The first is that we used import data to estimate national-level consumption as recommended by WHO [18]. The second is the use of standardized methodology which allows for comparisons over time and across countries. This approach has also proven to be a viable option for routine surveillance monitoring of antibiotic consumption in the Sierra Leone context, which is a priority for the National Strategic Plan for Combating AMR [17]. Third, all data transferred for inclusion in the analysis were cross-validated by the Principal Investigator. Fourth, the conduct and reporting of the study followed the Strengthening of Reporting of Observational Studies in Epidemiology (STROBE) guidelines [37].

However, there were limitations. First, using import data as a proxy for consumption required certain assumptions. We only included antibiotics that came through the recognized legal channels (the Queen Elizabeth II Quay and the Lungi International Airport). This does not include illegal or unofficial imports which are not registered with PBSL. In addition, the use of import data does not account for products which may be destroyed after the expiry date or which are otherwise not consumed. Antibiotics intended for human consumption may be used in the animal sector; our data did not include information about human or animal consumption, but assumed human consumption based on strength, type and route of administration. Finally, some antibiotics imported for use in Sierra Leone may be smuggled out to neighboring countries, which is not accounted for in this study.

Second, the standardized methodology came with limitations. We could not assign ATC codes and DDD values to certain antibiotics in the database, as they are yet to be coded by the ATC system, and, thus, were not included in the generation of the DDDs reported. In addition, under the AWaRe classification, some antibiotics yet to be assigned to the three categories had to be coded as ‘other’. While the methodology for DDD per 1000 inhabitants per day accounts for population size, its interpretation should be contextualized to the burden of disease, health care system and financing, and informal market share in the country, which may limit conclusions drawn from cross-country comparisons.

Third, the study relied on the completeness of information in the Drug Clearance and Importation Permits Database and the Drug Registration Records. The study data quality assurance processes revealed weaknesses in the design and use of paper-based forms and the electronic database. For example, for some products, validating the strengths and route of administration was based on information obtained from the product available in the market. Furthermore, we were unable to look at antibiotic consumption year-by-year because of challenges with the import date variable in the Drug Clearance and Importation Permits Database.

Despite the limitations, our study points to some important recommendations for policy and practice at the global and national levels. At the global level, the WHO should ensure that the ATC/DDD system accounts for antibiotic substances that are commonly utilized in LMICs, so that these products can be included in the standard calculation of DDDs. Similarly, the WHO should ensure that all commonly utilized antibiotic substances are included in the AWaRe classification system.

At the national level, this study identifies the need to improve information systems at PBSL, in terms of completeness and accuracy of data currently collected. This can be achieved through more comprehensive inclusion of key variables and also staff capacity building. The Sierra Leone AMR Technical Coordination Committee, which includes representation from the MoHS, PBSL, and WHO Country Office, amongst others, is well placed to support improvements in the PBSL information system, including the data capture and storage approach and staff capacity. Finally, Reserve antibiotics should be included in the National Standard Treatment Guidelines and be considered for the Essential Medicines List (EML) [22]. It is essential that these steps be complemented by the establishment of functioning antibiotic stewardship programmes and the strengthening of laboratory capacity for AMR in Sierra Leone.

## 5. Conclusions

Between 2017 and 2019, national antibiotic consumption for Sierra Leone was estimated at 19 DDD per 1000 inhabitants per day. Most antibiotic consumption was oral with parenteral use being less than 2%. Almost two-thirds of antibiotics consumed were in the Access AWaRe category, and there were no antibiotics consumed in the Reserve category. Finally, half of all the oral antibiotics consumed in Sierra Leone were metronidazole and ciprofloxacin, while half of all parenteral antibiotics were procaine benzylpenicillin and ceftriaxone. Policy recommendations at the global and national level have been made to help improve the monitoring of antibiotic consumption and antibiotic stewardship.

## Figures and Tables

**Table 1:** Total antibiotic consumption (J01) per defined daily dose (DDD) per 1000 inhabitants per day according to the anatomical, therapeutic, and chemical (ATC level 3) classification system, by route of administration, in Sierra Leone, 2017–2019.

Therapeutic/Pharmacological Subgroup (ATC3)	DDD per 1000 Inhabitants per Day					
	Total		Oral		Parenteral	
		(%)		(%)		(%)
J01A Tetracyclines	0.9	(5.1)	0.9	(5.2)	0.0	(0.0)
J01B Amphenicols	0.1	(0.6)	0.1	(0.6)	0.0	(0.7)
J01C Beta-lactam antibacterials/penicillins	2.9	(15.5)	2.7	(15.0)	0.1	(45.3)
J01D Other beta-lactam antibacterials	1.2	(6.2)	1.1	(6.0)	0.1	(19.4)
J01E Sulfonamides-trimethoprim	2.2	(11.7)	2.2	(11.8)	0.0	(0.0)
J01F Macrolides, lincosamides and streptogamins	1.9	(10.4)	1.9	(10.6)	0.0	(0.0)
J01G Aminoglycosides	0.0	(0.2)	0.0	(0.0)	0.0	(14.7)
J01M Quinolones	2.7	(14.7)	2.7	(14.9)	0.0	(3.4)
J01R Combinations	0.2	(1.0)	0.2	(1.0)	0.0	(0.0)
J01X Other antibacterials ^1^	6.4	(34.6)	6.3	(34.9)	0.1	(16.6)

^1^ Includes Metronidazole (P01AB01) and Tinidazole (P01AB02). J01: antibacterials for systemic use, ATC3: Anatomic, Therapeutic and Chemical classification, level 3 (therapeutic/pharmacological subgroup), DDD: defined daily dose.

**Table 2:** Total consumption of antibiotics (J01) per defined daily dose (DDD) per 1000 inhabitants per day by WHO AWaRe system in Sierra Leone, 2017–2019.

WHO AWaRe Category	DDD per 1000 Inhabitants per Day					
	Total		Oral		Parenteral	
		(%)		(%)		(%)
Access	12.0	(64.8)	11.8	(64.6)	0.2	(77.2)
Watch	5.7	(30.7)	5.6	(30.9)	0.1	(22.8)
Reserve	0.0	(0.0)	0.0	(0.0)	0.0	(0.0)
Other	0.8	(4.5)	0.8	(4.6)	0.0	(0.0)

**Table 3:** Top ten most commonly used oral antibiotics (J01, by ATC5) in relation to DDD per 1000 inhabitants and their percentage of all oral antibiotics consumed in Sierra Leone, 2017–2019.

Antibiotic Substance (ATC5 Code)	DDD per 1000 Inhabitants per Day	Percent of All Oral Antibiotics Consumed
Metronidazole (P01AB01)	6.3	34.9
Ciprofloxacin (J01MA02)	2.7	14.6
Sulfamethoxazole and trimethoprim (J01EE01)	2.2	11.8
Erythromycin (J01FA01)	1.9	10.6
Amoxicillin (J01CA04)	1.7	9.4
Tetracycline (J01AA07)	0.9	5.2
Cefuroxime (J01DC02)	0.9	5.1
Combinations of penicillins (J01CR50)	0.6	3.5
Amoxicillin and enzyme inhibitor (J01CR02)	0.2	1.0
Ampicillin (J01CA01)	0.2	0.9

J01: antibacterials for systemic use, ATC5: Anatomic and Therapeutic Classification, level 5 (chemical substance group), DDD: defined daily dose.

**Table 4:** Top ten most commonly used parenteral antibiotics (J01, ATC5) by DDD per 1000 inhabitants and their percentage of all parenteral antibiotics consumed in Sierra Leone, 2017–2019.

Antibiotic Substance (ATC5 Code)	DDD per 1000 Inhabitants per Day	Percent of All Parenteral Antibiotics Consumed
Procaine benzylpenicillin (J01CE09)	0.19	31.7
Ceftriaxone (J01DD04)	0.06	19.4
Metronidazole (J01XD01)	0.05	16.6
Gentamicin (J01GB03)	0.04	14.7
Benzylpenicillin (J01CE01)	0.03	9.7
Ciprofloxacin (J01MA02)	0.01	3.4
Ampicillin (J01CA01)	<0.01	2.2
Benzathine benzylpenicillin (J01CE08)	<0.01	1.6
Chloramphenicol (J01BA01)	<0.01	0.7
Amoxicillin (J01CA04)	<0.01	<0.1

J01: antibacterials for systemic use, ATC5: Anatomic, Therapeutic and Chemical classification, level 5 (chemical substance group), DDD: defined daily dose.

## Data Availability

The data that supports the finding of this study are available from the corresponding author J.S.K. upon a reasonable request.
